# Trace Analyses of Impurities in Povidone by Square Wave Voltammetry

**DOI:** 10.6028/jres.093.128

**Published:** 1988-06-01

**Authors:** Robert M. Ianniello

**Affiliations:** GAF Chemicals Corporation, Analytical Research & Development, 1361 Alps Road, Wayne, NJ 07470

Povidone (polyvinylpyrrolidone) is a water soluble polymer used in a variety of pharmaceutical, beverage, cosmetic, and agricultural applications. The free radical-induced polymerization of vinylpyrrolidone using aqueous hydrogen peroxide/ammonia as an initiator allows for rapid, easily controlled reactions. However, the reactivity of the various components can give rise to trace levels of undesirable by-products. The analytical methods available for these impurities utilize dissimilar procedures with time consuming sample preparation steps; and the need for high product quality requires methods with low detection limits for which many of the established procedures are not suitable.

A general approach for the analysis of impurities in our laboratory has involved the use of square wave voltammetry (SWV) for the quantitation of electroactive species. This technique couples the ability to determine species at very low levels (sub ppm) with analysis speed, making it ideal for aiding in process optimization. The version of square wave voltammetry used in our laboratory was developed by Osteryoung [1] and first commercialized by Bioanalytical Systems. In the technique, a pulse train of square waves is superimposed on a staircase wave form. A single square wave cycle (forward and reverse pulse) is generated per staircase step. Current is sampled near the end of each forward and reverse pulse. The difference in currents generated from the forward/reverse signals yields the analytical current response. The very nature of the experiment results in the following advantages:

- Scan rate, which is dependent primarily on square wave frequency, is fast. A typical experiment covering a potential range of 1 V may take only 5 seconds by SWV compared to 3 minutes by other pulse voltammetric techniques.
- For reversible systems, enhanced peak currents are obtained as the net current is the difference between a positive forward current and a reverse current which is *opposite* in sign.
- As a pulse technique, discrimination against charging current improves the detection limit.
- Multiple scanning is used with signal averaging to improve the signal-to-noise ratio.
- Diagnostic information can be obtained (as in cyclic voltammetry) by observing the individual forward and reverse currents.

Two impurities which have been analyzed successfully by the technique are acetaldehyde [2] and ammonium ion [3]. In the first case, acetaldehyde is irreversibly reduced at the mercury electrode in basic media. This allows for its direct detection via SWV. In the second case, ammonium ion is rendered electroactive by derivatization with formaldehyde to form hexamethylene tetramine. The derivative is then reduced in acid media at the mercury electrode. In both cases, superior sensitivity and detection limits are obtained, compared to conventional techniques.
